# Metastatic Lung Adenocarcinoma Harboring an *EGFR*-Activating Mutation in a Heart Transplant Recipient

**DOI:** 10.1200/JGO.17.00029

**Published:** 2017-06-30

**Authors:** Elizabeth Zambrano Mendoza, Cheng Tzu Yen, Tiago Kenji Takahashi, Gustavo Faibischew Prado, Marcelo Luiz Balancin, Gilberto de Castro

**Affiliations:** **Elizabeth Zambrano Mendoza**, **Cheng Tzu Yen**, **Tiago Kenji Takahashi**, **Gustavo Faibischew Prado**, and **Gilberto de Castro Jr,** Instituto do Câncer do Estado de São Paulo and Universidade de São Paulo; **Cheng Tzu Yen** and **Gilberto de Castro Jr**, Hospital Sírio Libanês; and **Marcelo Luiz Balancin**, Diagnóstika, São Paulo, Brazil.

## CASE REPORT

A 68-year-old man, a former smoker (35 pack-years) with an Eastern Cooperative Oncology Group performance status of 1, received heart transplantation for dilated idiopathic cardiomyopathy in 2012, followed by continuous immunosuppressive treatment with tacrolimus 2 mg/day and mycophenolate mofetil 720 mg/day.

**Fig 1:**
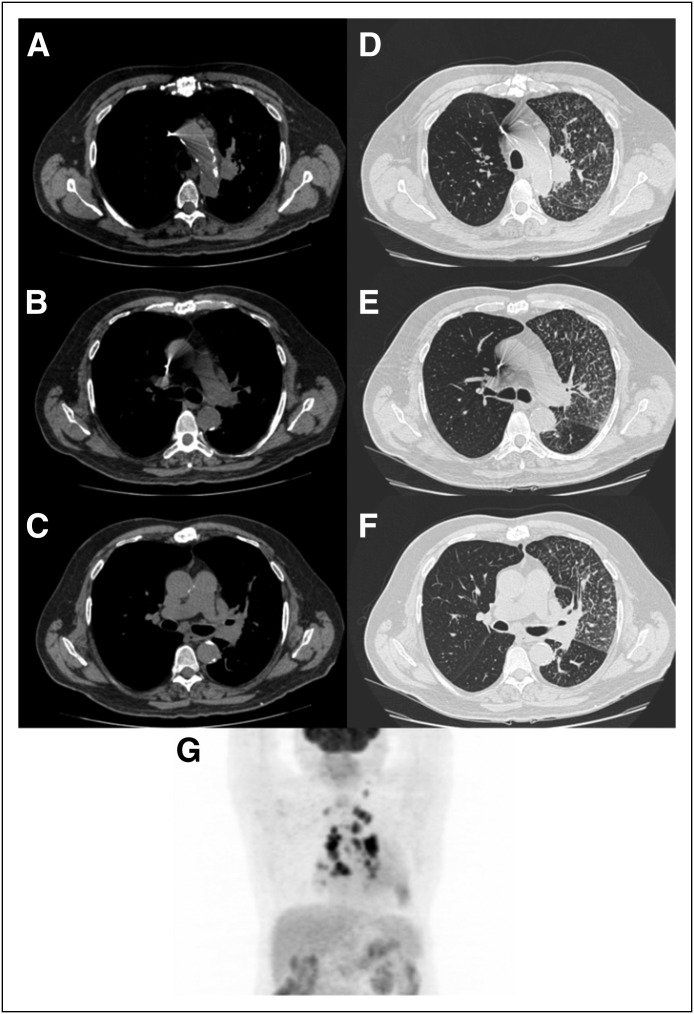
Computed tomography scan showing (A) left upper lung mass para aortic, (B) right paratracheal and (C) subcarinal (D) enlarged lymph nodes with nodular interlobular septal thickening compatible with lymphangitis carcinomatosa (D-F). ^18^F-labeled fluorodeoxyglucose (FDG) –positron emission tomography (maximum intensity projection reconstruction) showing both mass and bilateral (hilar and mediastinal) lymph nodes with intense FDG uptake, as well as the diffuse elevated uptake in the left lung.

In March 2016, this patient presented with symptoms of cough, weight loss, hyporexia, and dyspnea. Initial work-up demonstrated a 3.1 × 2.3–cm posterior left upper lobe mass, multiple bilateral lung micronodules, as well as several enlarged lymph nodes (ipsilateral hilum, bilateral upper paratracheal, and para-aortic sites). Pulmonary transbronchial biopsy revealed an adenocarcinoma (Fig 1). ^18^F-labeled fluorodeoxyglucose positron emission tomography showed increased uptake in primary mass, enlarged lymph nodes, and lymphangitic carcinomatosis (T2aN3M1a).

**Fig 2:**
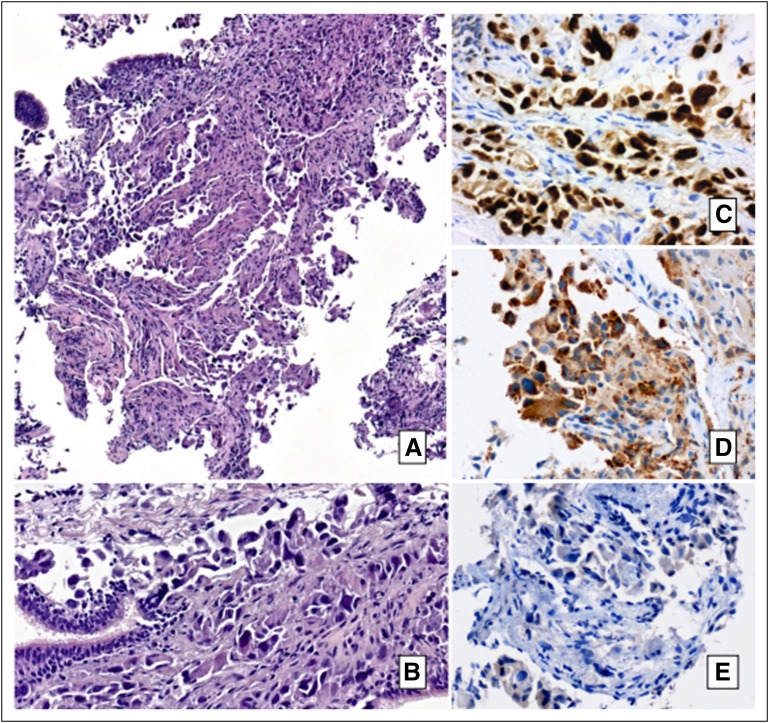
Pathology findings. (A-B) Low- and high-power images of hematoxylin and eosin (HE) –stained section from lung biopsy reveals a malignant epithelial neoplasm composed of atypical cells infiltrating lung tissue, consistent with non-small cell carcinoma (HE, x5, x40). (C-D) Immunohistochemistry (IHC) revealed TTF1 and napsin A strongly positive, supporting the diagnosis of lung adenocarcinoma (IHC, x40). (E) Anti-ALK IHC (negative) evaluated through D5F3 antibody.

A next-generation sequencing gene panel (TruSight 26-gene panel; Illumina, San Diego, CA) revealed a deletion in exon 19 of *EGFR* (p.Glu746_Ala750del), with no abnormalities in *ERBB2*, *BRAF*, *KRAS*/*NRAS*, *MET*, or *PIK3CA*; ALK translocations were not detected by immunohistochemistry (Fig 2).

**Fig 3:**
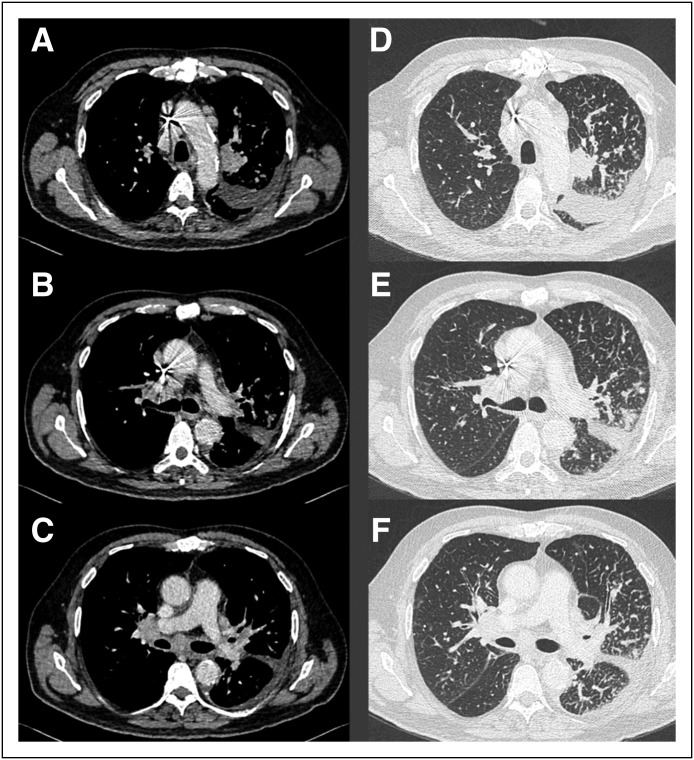
Computed tomography scan showing progressive disease characterized by bilateral mediastinal and hilar lymph node enlargement and pleural effusion (A-C), as well as progression of lymphangitis carcinomatosa (D-F).

This patient was administered erlotinib 150 mg/day concomitant with his immunosuppressant medications. The treatment was well tolerated, with only grade 1 skin toxicity after 3 weeks of erlotinib administration. After 5 weeks of treatment with erlotinib, the patient developed left pleural effusion, requiring thoracentesis and pleurodesis with immediate clinical improvement. After 7 weeks, a computed tomography scan showed progressive pleural, parenchymal, and mediastinal disease; chemotherapy with carboplatin and pemetrexed was started (Fig 3). No dose reduction or discontinuation was required. As of this writing, the patient presented with a partial response to chemotherapy and is receiving pemetrexed maintenance therapy.

## DISCUSSION

Recipients of solid organ transplant have an overall two- to three-fold increase in risk of a wide spectrum of malignancies compared with the general population, mainly as a result of the administration of immunosuppressive drugs for a prolonged period and the detrimental impacts on cancer immunosurveillance and potential direct carcinogenic effects.^1^ The incidence of post-transplant malignancies is approximately 4%^2^ and they account for 10% to 47% of deaths in solid organ recipients.^2^

Lung cancer after solid organ transplantation seems to occur at a higher frequency than in the nontransplanted population, especially after heart transplantation.^3^ Several studies have described potential risk factors (eg, age at transplantation, older age, male sex, smoking history, use and time of immunosuppression).^4^ It is estimated that the mean interval between transplantation and diagnosis is approximately 35 months. One-year and 3-year survival after cancer diagnosis were estimated as 60% and 52%, respectively.^5^

The management of advanced lung cancer in transplant recipients is complex as a result of many factors, such as the lack of cancer immunosurveillance, potential increased susceptibility to infections in patients receiving both chemotherapy and immunosuppressive treatments, unknown pharmacokinetic/pharmacodynamic interactions between oral tyrosine kinase inhibitors (TKIs) and immunosuppressive agents, and comorbidities. In addition, no large studies have established the safety and efficacy of epidermal growth factor receptor (EGFR)-TKIs in patients receiving immunosuppressive therapy, and those patients are usually excluded from clinical trials. The pharmacokinetic/pharmacodynamic effects of the combination of erlotinib (as well as other EGFR TKIs) and immunosuppressive agents are not well documented. However, it is recognized that CYP3A4 has an important role in the metabolism of erlotinib; likewise, CYP3A4-mediated metabolism occurs with tacrolimus, with potential increases or decreases in plasma levels of these drugs. As a consequence, clinically significant variations in plasma levels can occur, leading to unexpected adverse effects or lack of efficacy of these drugs. In this case, we did not check plasma concentrations of erlotinib as a possible explanation for disease progression after only 2 months in a patient with an *EGFR* deletion in exon 19.

In conclusion, recipients of heart transplant must be followed accordingly with chest computed tomography (annually), which can also serve as a screening procedure for chest malignancies, considering the increased risk for those cancers. If metastatic lung cancer is diagnosed, the patient’s work-up should follow current recommendations, including molecular studies to diagnose driver mutations in *EGFR* and *ALK*. Monitoring plasma levels of immunosuppressive drugs should be considered to better understand the potential drug interactions in these patients.
